# Effectiveness of simulation-based clinical research curriculum for undergraduate medical students** - **a pre-post intervention study with external control

**DOI:** 10.1186/s12909-024-05455-6

**Published:** 2024-05-15

**Authors:** Qiao Huang, Si-Yu Yan, Jiao Huang, Yi Guo, Xian-Tao Zeng, Ying-Hui Jin

**Affiliations:** 1 Center for Evidence-Based and Translational Medicine, Zhongnan Hospital of Wuhan University, #169, East Lake Road, Wuchang District, Wuhan City, Hubei Province China; 2 Department of Evidence-Based Medicine and Clinical Epidemiology, Second School of Clinical Medicine, Wuhan University, Wuhan, 430071 China; 3 Department of Geriatrics, Zhongnan Hospital of Wuhan University, Wuhan, 430071 China

**Keywords:** Medical student, Clinical research, Simulation teaching, Curriculum evaluation, Curricular innovation, Pre-post study, External control

## Abstract

**Background:**

Simulation is widely utilized in medical education. Exploring the effectiveness of high-fidelity simulation of clinical research within medical education may inform its integration into clinical research training curricula, finally cultivating physician-scientist development.

**Methods:**

Standard teaching scripts for both clinical trial and cross-sectional study simulation were designed. We recruited undergraduates majoring in clinical medicine at 3^th^ grade into a pre-post intervention study. Additionally, a cross-sectional survey randomly selected medical undergraduates at 4^th^ or 5^th^ grade, medical students in master and doctor degree as external controls. Self-assessment scores of knowledge and practice were collected using a 5-point Likert scale. Changes in scores were tested by Wilcoxon signed-rank test and group comparisons were conducted by Dunn’s tests with multiple corrections. Multivariable quantile regressions were used to explore factors influencing the changes from baseline.

**Results:**

Seventy-eight undergraduates involved the clinical trial simulation and reported improvement of 1.60 (95% CI, 1.48, 1.80, *P* < 0.001) in knowledge and 1.82 (95% CI, 1.64, 2.00, *P* < 0.001) in practice score. 83 undergraduates involved in the observational study simulation and reported improvement of 0.96 (95% CI, 0.79, 1.18, *P* < 0.001) in knowledge and 1.00 (95% CI, 0.79, 1.21, *P* < 0.001) in practice. All post-intervention scores were significantly higher than those of the three external control groups, *P* < 0.001. Higher agreement on the importance of clinical research were correlated with greater improvements in scores. Undergraduates in pre-post study showed high confidence in doing a future clinical research.

**Conclusion:**

Our study provides evidence supporting the integration of simulation into clinical research curriculum for medical students. The importance of clinical research can be emphasized during training to enhance learning effect.

**Supplementary Information:**

The online version contains supplementary material available at 10.1186/s12909-024-05455-6.

## Introduction

Clinical research is a key component of medical researches which recruits human beings as participants. It aims at generating valuable and insightful knowledge in understanding disease mechanisms, preventing and treating diseases, and promoting health [1]. Common types of clinical research included interventional study (e.g. randomized controlled trial, RCT) and observational study (e.g. cohort study, case–control study and cross-sectional study, et. al). Medical students are individuals who are enrolled in a medical school and are undergoing a structured program of theoretical coursework and practical clinical training (clerkship student) to become qualified physicians [2]. Medical students should not only master how to manage patients, but also study clinical research skills, which have been recognized domestically and internationally [3, 4]. The Association for Medical Education in Europe (AMEE) emphasized the importance of research skills and its related attributes for medical students pursuing a medical career and developed professional guidance [3]. Learning and engagement in clinical researches allow medical students to cultivate their ability of critical thinking, innovation and scientific research, and develop academic careers as independent clinical investigators. Furthermore, medical students will directly participate in medical practice and need to apply scientific evidences to optimize patient diagnosis and treatment in the future. Clinical research serves as a catalyst for medical students to embrace evidence-based practice (EBP) in patient care by bridging the gap between knowledge in classroom and real-world clinical applications [5, 6]. Ultimately, this process contributes to the development of well-rounded physician-scientists [7]. However, many physicians lacked the skills necessary to understand and conduct clinical research to an international standard, primarily due to the absence of systematic training in clinical research within medical education institutions [8]. So, systematically training medical students in clinical research is of significant importance.

In medical education, high theory score didn’t guarantee high practical ability [9]. Simulation-based medical education (SBME) uses a variety of simulation methods to create a real-world clinical scenario, such as role play, virtual reality simulation and task-oriented and mannequin-based simulation [10]. SBME allows students to engage in realistic clinical scenarios and gain hands-on experience in a safe, standardized and interactive learning environment. Moreover, teams are always built in SBME, which can also train students’ cooperation, communication, leadership and others vital for medical care. A growing body of evidence suggested that SBME was effective for teaching clinical knowledge, skills, and behaviors compared with traditional methods. Studies have found the effect of SBME in cardiology [11], anesthesiology [12], and anatomy [13]. It might be beneficial to use SBME in clinical research curriculum. By mirroring the real-world clinical research process, research skills can be trained, including protocol design, recruitment, data collection and analysis, report writing and dissemination. Previous studies employed simulation in protocol development [14], principles and concepts of outbreak [15], research design [5, 16]. However, comprehensive evidences on effectiveness of high-fidelity simulation for overall process of clinical researches is still limited.

To address the existing gap, we have systematically developed a novel simulation-based clinical research curriculum framework [17]. In this study, we apply a pre-post intervention study on undergraduate medical students with external controls to quantitatively the effectiveness of SBME for clinical research training. Our research aims to contribute evidences that may shape the integration of high-fidelity simulation into the medical education landscape for clinical research training.

## Methods

### Study oversight

**Fig. 1:**
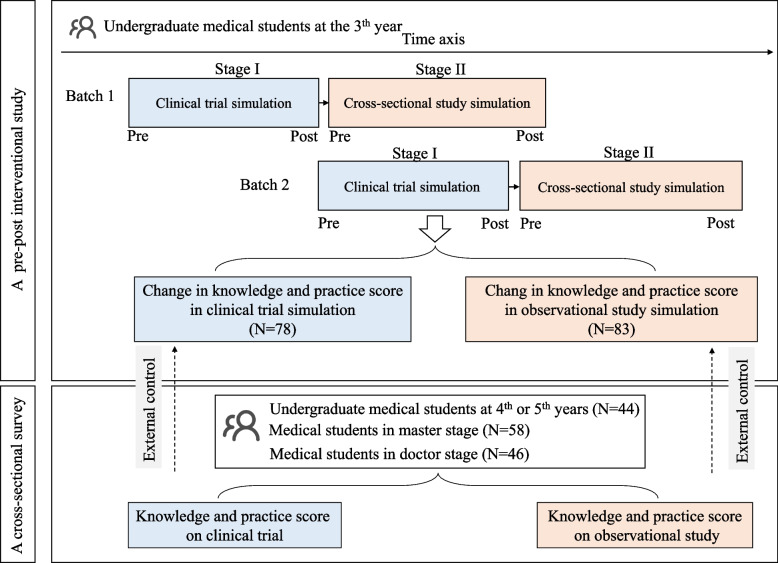
Flowchart for study design

This study comprised a prospective pre-post intervention study and a cross-sectional survey (Fig. 1). It was conducted between 2020 and 2023 in a medical college of a prominent university in Wuhan city, China. It was approved by the medical ethics committee of Zhongnan Hospital of Wuhan University (Approved number of ethic committee: 2020111 K). Medical students were recruited as participants, and online questionnaires were distributed among them. Clicking the ‘Agree’ button signified the students’ consent to participate in this study.

### Pre-post intervention study

Due to ethical considerations, a RCT was not feasible. Consequently, a single-arm study with pre-post measurements was conducted on the same group of participants to evaluate the potential effect of SBME on clinical research training. In this study design, no random allocation, allocation concealment, or blinding was employed.

#### Participants

Undergraduate medical students at 3^rd^ grade were recruited as participants to sequentially attend both RCT simulation and observational study simulation. The pre-post study had two student cohorts, including batch 1 (year 2020–2021) and batch 2 (year 2021–2022), respectively (Fig. 1). Participants who agreed to participate and reported scores both prior to and following the simulation curriculum were included for further analysis.

#### Course development and intervention implementation

The pre-post intervention encompassed simulation-based curricula for both RCT scenario and cross-sectional study (observational study) scenario. The simulation-based curricula aimed to provide medical students with theoretical basics and practical skills for participating in clinical research projects with their tutors in the future. About twenty faculty members with expertise in clinical study and evidence based medicine, including all authors, engaged in multiple rounds of discussions to construct the curriculum framework and supporting materials based on the adapted Kern’s six-step model. The curriculum content was further reviewed and approved by the course administrators. Development process and teaching scripts have been comprehensively detailed [17].

All recruited students participated in a two-stage course (Fig. 1). Stage I was the clinical research 1 (simulation for clinical trial) during the 5^th^ semester, stage II was the clinical research 2 (simulation for cross‑sectional study) during the 7^th^ semester. During the two semesters, medical students were also enrolled in both foundational theoretical and professional medical courses. The clinical research 1 curriculum was a simulation based on a published clinical trial of Remdesivir in adults with severe COVID-19 [18]. The clinical research 2 curriculum was an observational-study simulation to investigate prevalence and risk factors of hypertension in Wuhan city, China. Both clinical research 1 and 2 included 11 classes. Comprehensive details, including the learning objectives, equipment, task-based simulation processes and time schedule, and required deliverables, were provided in the Supplementary files 4 and 5 of our previous work [17].

All authors were involved in teaching processes of the two simulation courses. Before the curriculum, students were provided with operation manual which facilitated a comprehensive understanding of the entire flow of the simulation curriculum. Each class is delivered through face by face instruction in a general university classroom. The teaching team included one primary instructor and four assistant instructors in class. Students were divided into groups of 6–8 and tables in classroom were arranged to facilitate group collaboration. Each class took about 135 min. The primary instructor commenced with a brief didactic session to cover essential concepts before hands-on practice. Subsequently, students reviewed the operation manual and participated in role-playing activities, assuming diverse roles including sponsors, monitors, ethics committee members, data collector and statisticians, to complete group-based assignments, such as developing a clinical trial protocol and compiling a clinical study report. Throughout the simulation, four assistant instructors roamed the area to ensure prompt responses to students’ queries and needs. At end of each class, the primary instructor invited students to share their experience and difficulties encountered during this simulation, meanwhile, the teacher provided solutions to the proposed problems and concluded the class with a summary.

#### Development of assessment tools

Two assessment questionnaires were designed to evaluate intervention effects following the RCT and cross-sectional study simulation courses, respectively. Each questionnaire included self-assessment of knowledge and practice part. Items in this part were supplied by the teachers of the simulation courses, who were required to ensure that these items accurately represented the key learning objectives of each class. Before formal distribution, two experts in epidemiology (YG) and clinical practice (XT-Z) reviewed and revised the contents of the two assessment tools to guarantee face validity. To assess test–retest reliability, a total of 20 medical students were randomly selected from the medical college, with 10 assigned to the questionnaire for the RCT simulation and 10 to the questionnaire for the cross-sectional study simulation. About fourteen days after the initial evaluation, the same 20 students were requested to complete corresponding questionnaires again. The test–retest reliability coefficients (intra-class correlation coefficients, ICC) were calculated as 0.764 for the RCT simulation questionnaire and 0.719 for the cross-sectional study simulation questionnaire, respectively. Furthermore, feedbacks were gathered from the 20 students to refine the final questionnaires.

In the pre-simulation questionnaire, basic characteristics were collected as covariates (Part 1 in Supplementary files 1 and 2), including sex (male/female), age (years), having participated in clinical research (yes/no), having received systematic training in clinical research (yes/no), proactive self-learning (yes/no), willing to conduct clinical research (yes/no) and having heard of simulation teaching (yes/no). Additionally, agreement level on “improving clinical research ability can improve the clinical practice ability of medical staff” and “clinical research can promote the development of medical science and technology, and ultimately benefit patients” were assessed by Likert scale from 0 (Strongly disagree) to 10 (Strongly agree).

In both pre- and post- simulation questionnaire, self-assessment of knowledge and practice were collected as primary outcomes for RCT and cross-sectional study simulations, respectively (Part 2 in Supplementary files 1 and 2). The assessment for RCT included 5 dimensions with 22 questions: (1) protocol development, (2) ethics application, (3) case report form, (4) randomization, blindness and recruitment and (5) unblinding, statistical analysis and interpretation. The assessment for observational study included 5 dimensions with 14 questions: (1) observational study protocol, (2) data collection tool and operation manual, (3) pre and formal survey and recruitment, (4) data collection and verification and (5) statistical analysis and interpretation. Each question was used to assess participants’ knowledge and practical ability, using a 5-point Likert scale (1 = Very unfamiliar; 2 = Unfamiliar; 3 = Moderate; 4 = Familiar; 5 = Very familiar). Mean scores for each dimension and the overall were calculated.

In the post-simulation questionnaire, participants were further required to fill post-curriculum assessment on SBME (Part 3 in Supplementary files 1 and 2). The assessment of effect of our curriculum incorporated items from a prior Chinese study that utilized scenario simulation to enhance the doctor-patient communication skills of resident physicians [19]. It measured the extent of agreement or disagreement with the following statements regarding the simulation teaching: (1) deepening the theoretical knowledge, (2) improving communication skills and abilities, (3) improving teamwork skills, (4) increasing learning interest, (5) improving critical thinking, (6) improving practical skills and (7) improving ability to handle emergencies in research, scoring from 1 (Strongly disagree) to 5 (Strongly agree). Given the reliance on teaching expertise, no alteration was made to these items. However, it still required further quantitative validation. Participants also reported level of agreement with the utilization of situational simulations in the clinical study training course, using a scale from 1 (Strongly disagree) to 5 (Strongly agree). Furthermore, they expressed their confidence levels in independently conducting clinical research in the future, with a range from 0 (no confidence) to 10 (high confidence).

#### Data collection

In either RCT simulation or cross-sectional study simulation, participating students were required to assess their knowledge and practice scores before and after the curriculum. The pre-simulation questionnaire had two parts (basic characteristics and self-assessment of knowledge and practice), and the post-simulation questionnaires included three parts (simplified basic characteristics, self-assessment of knowledge and practice, and assessment on SBME). These questionnaires were adapted into an online format with corresponding QR (quick-response) codes (QH). SY-Y and JH were responsible for contacting class monitor to distribute the online QR codes within the participants’ online contact group (WeChat group and QQ group) both before and after the overall simulation curricula. A total of 78 undergraduates took part in the RCT simulation, with 33 participants from Batch 1 and 45 participants from Batch 2. Meanwhile, there were 83 undergraduates involved in the observational study simulation, comprising 41 participants in Batch 1 and 42 participants in Batch 2.

### Cross-sectional survey as external controls

The absence of a contemporaneous control group in the previous pre-post intervention study complicates the attribution of changes in outcomes to the SBME intervention versus natural progression over time. External control would provide a comparative benchmark and a reference point to evaluate the effectiveness and generalizability of the SBME intervention. In this study, a cross-sectional survey was conducted in June 2023 to provide external controls for previous pre-post intervention study. A total of 148 medical students from the same college who had not attended our simulation curricula were randomly recruited. It consisted of 44 undergraduates at 4^th^ or 5^th^ grade, 58 medical students in master degree, and 46 medical students in doctor degree. A modified questionnaire was created, including basic characteristics (part 1), self-evaluation of knowledge and practical ability in RCT (part 2) and observational study (part 3), seen in Supplementary file 3. QH and JH contacted the monitor of medical college to distribute the corresponding online questionnaire.

### Statistical analysis

In descriptive statistics, categorical variables were described using count (%). The Likert scale was summarized using median (25^th^—75^th^ percentile). Group comparisons in paired samples and independent samples, quantile regressions were conducted as inferential statistics. All analyses were carried out using the R (version 4.2.1, R Foundation for Statistical Computing, Vienna, Austria). Two-sided *P* values below 0.05 were considered statistically significant.

#### Pre-post intervention study

Sample size calculation was conducted by G-Power version 3.1, using a two-sided alpha level of 0.05, a power of 90% and the statistical method of Wilcoxon signed-rank test. To observe an average elevation of 1 score from baseline in a Likert scale of 5 points with a standard deviation of 2, a sample size of 47 participants was required. Assuming a loss to follow-up rate of 20%, finally, at least 59 students should be recruited. Changes in knowledge and practice scores were tested by Wilcoxon signed-rank test, corresponding 95% confidence intervals (CIs) were reported. Multivariable quantile regression was used to explore independent effects of basic characteristics on change in scores at 50^th^ quantile, coefficients and 95% CIs were presented.

#### Cross-sectional survey as external controls

Knowledge and practice scores of undergraduates at 4^th^ or 5^th^ grade, medical students in master and doctor degree from cross-section part were compared with baseline and post-intervention scores of undergraduates from the interventional study. Dunn’s test using rank-sums was employed for multiple comparison, adjusted *P* values were reported to account for multiplicity.

## Results

### Sample characteristics

In pre-post intervention study, there were 78 participants for evaluating effect of RCT simulation and 83 participants for observation study simulation (Fig. 1 and Table 1). Meanwhile, a total of 148 participants from the cross-sectional survey were designated as external controls. Basic characteristics of these participants were summarized in Table 1. About half of participants were males and average age was about 21. In the pre-post part, 7 (4.35%) individuals had previous experience in clinical studies, and 21 (13.04%) participants had received systematic training related to clinical studies. The rates increased to 36.49% and 37.84% in participants from cross-sectional survey. Across all participants, there was a high level of agreement concerning the importance of clinical research in clinical practice and development, with median scores of 8 and 9, respectively.

**Table 1:** Basic characteristics of participants

Characteristics	Pre-post intervention part (simulation)		Cross-sectional survey (*N* = 148)
	Clinical trial (*N* = 78)	Observational study (*N* = 83)	
Batch			
Batch 1	33(42.31%)	41(49.40%)	-
Batch 2	45(57.69%)	42(50.60%)	-
Sex, male, n(%)	36(46.15%)	37(44.58%)	71(47.97%)
Age, median (min, max)	20 (19, 21)	21(20, 23)	24(23, 27)
Have participated in, n(%)	4(5.13%)	3(3.61%)	54(36.49%)
Have received systematic training in, n(%)	8(10.26%)	13(15.66%)	56(37.84%)
Proactive self-learning, n(%)	31(39.74%)	30(36.14%)	-
Willing to conduct, n(%)	74(94.87%)	74(89.16%)	-
Average agreement	8.5(7.5, 10.0)	8.0 (7.5, 10.0)	8.0(7.5, 9.75)
Level of agreement 1	8.0(7.0, 10.0)	8.0(7.0, 10.0)	8.0(7.0, 10.0)
Level of agreement 2	9.0(8.0, 10.0)	9.0(8.0, 10.0)	9.0(8.0, 10.0)
Have heard of simulation teaching, n(%)	36(46.15%)	-	-

Agreement 1, Do you agree that “Improving clinical research ability can improve the clinical practice ability of medical staff”?

Agreement 2, Do you agree that “Clinical research can promote the development of medical science and technology, and ultimately benefit patients”?

Agreement 1 and 2 range from 0 (Strongly disagree) to 10 (Strongly agree)

*RCT* randomized controlled trial

### Effectiveness of simulation-based curriculums and its influence factors

Knowledge and practice scores before and after the curriculum, along with the corresponding changes (95% CIs), have been summarized in Table 2. In both clinical trial and observational study simulations, there were significant increases in overall knowledge and practice scores, as well as their individual dimensions (all *P* < 0.001). In clinical trial simulation, participants reported the highest improvement in both knowledge and practice score for “development of case report form” and “Unblinding, statistical analysis and interpretation” dimensions. Meanwhile, the highest improvement was observed in the “data collection and verification” dimension in observational study simulation.

**Table 2:** Change in knowledge and practice scores from pre-intervention to post-intervention for clinical trial and observational study simulations

Domains	Knowledge			Practice		
	Before M (Q1, Q3)	After M (Q1, Q3)	Change (95% CI)^a^	Before M (Q1, Q3)	After M (Q1, Q3)	Change (95% CI)^a^
Simulation on clinical trial						
**Overall**	**2.1 (1.8, 2.9)**	**4.0 (3.6, 4.1)**	**1.6(1.5, 1.8)**	**1.9 (1.3, 2.3)**	**3.8 (3.3, 4.1)**	**1.8(1.6, 2.0)**
Protocol development	2.5 (2.0, 3.0)	4.0 (3.5, 4.0)	1.5(1.3, 1.5)	2.0 (1.0, 2.5)	3.8 (3.0, 4.0)	1.8(1.5, 2.0)
Ethics application	2.3 (1.8, 3.0)	4.0 (3.3, 4.0)	1.5(1.4, 1.8)	2.0 (1.3, 2.7)	4.0 (3.0, 4.0)	1.8(1.5, 1.9)
Case report form	2.0 (1.6, 2.8)	4.0 (3.8, 4.0)	1.8(1.6, 2.0)	2.0 (1.1, 2.2)	4.0 (3.2, 4.0)	1.9(1.7, 2.0)
Randomization, blindness and recruitment	2.3 (2.0, 3.0)	4.0 (3.8, 4.2)	1.5(1.3, 1.7)	2.0 (1.2, 2.5)	4.0 (3.5, 4.0)	1.8(1.7, 2.0)
Unblinding, statistical analysis and interpretation	2.0 (1.5, 2.6)	4.0 (3.4, 4.0)	1.8(1.6, 2.0)	2.0 (1.0, 2.2)	4.0 (3.0, 4.0)	1.9(1.7, 2.1)
Simulation on observational study						
**Overall**	**2.9 (2.4, 3.2)**	**3.9 (3.3, 4.0)**	**1.0 (0.8, 1.2)**	**2.6 (2.0, 3.1)**	**3.6 (3.1, 4.0)**	**1.0 (0.8, 1.2)**
Protocol development	3.0 (2.7, 3.3)	3.7 (3.0, 4.0)	1.0 (0.7, 1.2)	3.0 (2.0, 3.0)	3.3 (3.0, 4.0)	1.0 (0.7, 1.2)
Data collection tool and operation Manual	3.0 (2.5, 3.5)	4.0 (3.5, 4.0)	1.3 (1.0, 1.5)	3.0 (2.0, 3.0)	4.0 (3.0, 4.0)	1.0 (1.0, 1.3)
Pre and formal survey and Recruitment	3.0 (2.4, 3.2)	4.0 (3.3, 4.0)	1.2 (1.0, 1.4)	2.8 (2.0, 3.0)	3.8 (3.0, 4.0)	1.1(0.9, 1.4)
Data collection and verification	3.0 (2.0, 3.0)	4.0 (3.0, 4.0)	1.5 (1.3, 1.5)	2.5 (2.0, 3.0)	3.5 (3.0, 4.0)	1.5 (1.3, 1.5)
Statistical analysis and interpretation	3.0 (2.0, 3.0)	4.0 (3.0, 4.0)	1.3 (1.0, 1.5)	2.0 (2.0, 3.0)	3.5 (3.0, 4.0)	1.5 (1.3, 1.5)

*M* median, *Q1* the first quartile, *Q3* the third quartile, *CI* confidence interval

^a^All *P* values of changes were less than 0.001

**Fig. 2:**
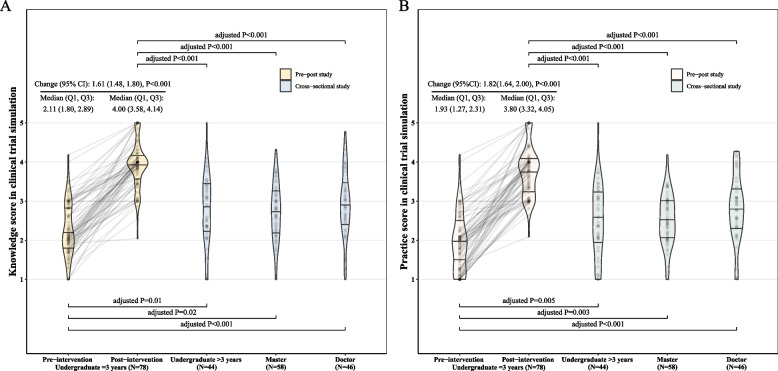
Change from baseline in clinical trial simulation and comparison with external controls

For clinical trial simulation, the median of overall knowledge score increased from 2.11 to 4.00 with a pre-post correlation of 0.35 (*P* = 0.002) and a change of 1.60 (95% CI, 1.48, 1.80, *P* < 0.001), seen in Fig. 2A. When comparing with external control, both pre-intervention and post-intervention knowledge scores were significantly different from the knowledge scores of undergraduates at more than 3rd grade (adjusted *P* = 0.01 and < 0.001), medical student in master degree (adjusted *P* = 0.02 and < 0.001) and those in doctor degree (adjusted *P* < 0.001 and < 0.001). Similarly, corresponding practice score increased from 1.93 to 3.80 with a change of 1.82 (95% CI, 1.64, 2.00, *P* < 0.001) and a pre-post correlation of 0.26 (*P* = 0.02), seen in Fig. 2B. Statistically significant differences of practice score were also identified among pre and post intervention groups and the three external control groups. The scores before the intervention were lower than those of the external controls, but after the intervention, the scores surpassed those of the external controls.

**Fig. 3:**
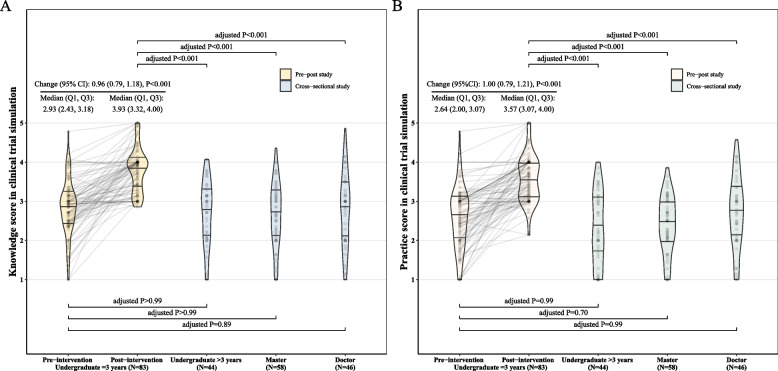
Change from baseline in cross-sectional study simulation and comparison with external controls

In the case of observational study simulation, the median of overall knowledge score increased from 2.93 to 3.93, resulting in a pre-post correlation of 0.22 (*P* = 0.049) and a change of 0.96 (95% CI, 0.79, 1.18, *P* < 0.001), seen in Fig. 3A. Additionally, the median of the overall practice score was elevated from 2.64 to 3.57, with a pre-post correlation of 0.17 (*P* = 0.12) and a change of 1.00 (95% CI, 0.79, 1.21, *P* < 0.001), as shown in Fig. 3B. Pre-intervention scores in both knowledge and practice were not significantly different from those of the three external controls. However, after the intervention, the corresponding scores were significantly higher than those of the three external control groups, with all *P* < 0.001.

Four multivariable quantile regressions were conducted to analyze changes in knowledge and practice scores for both clinical trial and observational study simulation (Table 3). All regressions consistently demonstrated that higher levels of agreement on the importance of clinical research were correlated with greater improvements from the baseline. The coefficients were 0.12 for clinical trial simulation and 0.13 for observational study simulation. In observational study simulation, we found batch effect in change in both knowledge and practice, beta = -0.43 (95% CI, -0.69, -0.17, *P* = 0.001) and -0.28 (95% CI, -0.57, 0.01, *P* = 0.06), respectively. Further group difference analysis showed batch 2 had higher baseline score in knowledge and practice than those of batch 1, *P* < 0.001 and 0.003, respectively. Additionally, a significant sex difference was identified in the change of practice scores for clinical trial simulation (adjusted difference = -0.42 [95% CI, -0.68, -0.16], *P* = 0.002).

**Table 3:** Influence of basic characteristics on changes in both knowledge and practice score from baseline using quantile regression $(\uptau =0.5)$

Characteristics	Clinical trial simulation				Observational study simulation			
	Change of knowledge		Change of practice		Change of knowledge		Change of practice	
	Beta (95% CI)	*P*	Beta (95% CI)	*P*	Beta (95% CI)	*P*	Beta (95% CI)	*P*
Baseline score	**-0.72 (-0.82, -0.62)**	** < 0.001**	**-0.66 (-0.81, -0.51)**	** < 0.001**	**-0.69 (-0.88, -0.50)**	** < 0.001**	**-0.80 (-1.00, -0.61)**	** < 0.001**
Batch								
2	-0.05 (-0.30, 0.20)	0.67	-0.01 (-0.29, 0.28)	0.96	**-0.43 (-0.69, -0.17)**	**0.001**	-0.28 (-0.57, 0.01)	0.06
1	ref		ref		ref		ref	
Sex								
Female	-0.21 (-0.50, 0.08)	0.16	**-0.42 (-0.68, -0.16)**	**0.002**	0.10 (-0.14, 0.34)	0.41	0.05 (-0.21, 0.30)	0.72
Male	ref		ref		ref		ref	
Age	-0.03 (-0.28, 0.21)	0.79	0.01 (-0.26, 0.28)	0.92	0.00 (-0.18, 0.18)	0.99	0.07 (-0.13, 0.28)	0.49
Not received systematic training	0.05 (-0.16, 0.26)	0.64	-0.15 (-0.57, 0.27)	0.48	-0.02 (-0.64, 0.60)	0.95	-0.10 (-0.75, 0.54)	0.75
Have no proactive self-learning	-0.14 (-0.38, 0.11)	0.26	-0.17 (-0.43, 0.10)	0.21	-0.06 (-0.30, 0.19)	0.64	0.07 (-0.21, 0.36)	0.61
Average agreement^a^	**0.12 (0.03, 0.20)**	**0.007**	**0.12 (0.01, 0.22)**	**0.03**	**0.13 (0.05, 0.20)**	**0.001**	**0.13 (0.05, 0.21)**	**0.001**
Not heard of simulation teaching	-0.01 (-0.25, 0.23)	0.94	0.08 (-0.19, 0.35)	0.56	-	-	-	-

*CI* confidence interval

^a^Average agreement regarding importance of clinical research on clinical practice and development

### Evaluation of the simulation curriculum after the intervention

The median score of evaluations for both clinical trial and observational study simulations across the 7 items was 4, indicating high perceived improvement in these skills (Table 4). Furthermore, participants reported high agreement on the use of situational simulations in both curriculums (median = 4), along with a strong confidence in their ability to independently conduct clinical research in the future (median = 8 for clinical trial and 7 for observation study).

**Table 4:** Evaluation on scenario simulation among participants in the pre-post intervention study

Items	Clinical trial simulation	Observational study simulation
Effect of simulation-based education^a^		
Deepen the mastery of theoretical knowledge	4.0(4.0, 5.0)	4.0(4.0, 5.0)
Improve communication skills and abilities	4.0(4.0, 5.0)	4.0(4.0, 5.0)
Improve teamwork skills	4.0(4.0, 5.0)	4.0(4.0, 5.0)
Increase learning interest	4.0(4.0, 5.0)	4.0(4.0, 5.0)
Improve critical thinking	4.0(4.0, 5.0)	4.0(4.0, 5.0)
Improve practical skills	4.0(4.0, 5.0)	4.0(4.0, 5.0)
Improve the ability to handle emergencies in research	4.0(4.0, 5.0)	4.0(4.0, 5.0)
Agreement with the use of situational simulations in the course^a^	4.0(4.0, 5.0)	4.0(4.0, 5.0)
Level of confidence to independently conduct a clinical study in the future^b^	8.0(6.0, 9.0)	7.0(7.0, 9.0)

^a^range from 1 (strongly disagree) to 5 (strongly agree)

^b^range from 0 (very unconfident) to 10 (very confident)

## Discussion

Simulation-Based Medical Education (SBME) is a teaching methodology for medical students and healthcare professionals that implements simulated environments and scenarios to mimic real-world clinical experiences. This study, consisting of a pre-post intervention study and a cross-sectional survey, showed that SBME for clinical research training enhanced medical students’ knowledge and practice. Moreover, medical students expressed their endorsement of the teaching method and improved confidence in conducting their future clinical research. Elevating medical students’ awareness of the importance of clinical research could further amplify the effectiveness of the SBME approach. The findings provided evidence to support the development and incorporation of SBME into clinical research curriculum.

Medical students should undergo systematic training before they independently apply their knowledge and experience in the provision of patient care for patient safety. Medical students are increasingly exposed to SBME, especially in their late years of study career [20]. SBME can improve medical students’ both knowledge and practical performance. Timely feedback from SBME can further provide opportunities to correct mistakes and strengthen learning experiences. However, a recent systematic review showed that most simulation studies focused on clinical skills and suggested future studies to broaden the scope of SBME [21]. Attention has been paid to the waste of resources caused by non-standard clinical research [22]. Clinical doctors play an indispensable role in conducting clinical research. Early experience in clinical and community settings benefited medical students [23]. Furthermore, early introduce of clinical research skills into the medical curriculum during undergraduate careers was also recommended [3, 24]. Timely and systematic training on clinical research abilities before carrying out clinical research can help them improve research quality and produce high-quality evidence.

Objective development and assessment is crucial to any curricular innovation prior to widespread implementation. The development process of our innovative simulation-based clinical research curriculum has been published, with a detailed teaching plan attached [17]. The Prescribed-Intended-Enacted-Sustainable (PIES) framework was proposed for evaluative research on implementation of curricular innovations [25]. We took the curriculum evaluation with reference to this guidance, especially in “enacted curriculum” and “sustainable curriculum” parts. Our study revealed significant improvement in students’ confidence and their acceptable satisfaction with the teaching method. Similar findings have been reported in studies focusing on high-fidelity simulation in clinical research training [14] and a surgical patient pathway simulation training [26]. The observed positive findings could be explained by the student-centered active learning and immersive environments [27]. While conducting simulation training of clinical skills, simulation-based clinical research curricula facilitate students’ development of essential research knowledge, skills and confidence. Organic integration of both aspects can effectively nurture proficient physician-scientist [28].

Clinical research is an important component of medical development, as it can generate evidence that directly guide clinical practice. As future clinicians, medical students should be informed that they can not only utilize existing evidence but also generate new evidence [29]. Our study showed that increased awareness of the importance of clinical research could better benefit medical students. SBME embodies a student-centered mindset and requires high engagement level of students. But this poses a challenge to students’ consciousness of learning. In practice, we encountered few students who act negatively in the class. Based on the Knowledge, Attitude, and Practice (KAP) theory, assisting students in comprehending the value of clinical research can enhance their interest and enthusiasm towards clinical research, making them more willing to engage in the simulation courses [30]. So, it is essential to establish a pervasive culture of clinical research within routine HPE environment. Moreover, the education can further encourage the application of EBP in students’ future clinical practices. Our team designed standard scripts for simulation-based clinical research course, however, heterogeneity in observational study simulation was observed in our study. It might partly be attributed to higher baseline scores in batch 2. Negative correlation between baseline and change from baseline might explain the lower change in batch 2 [31]. To ensure the benefit for all students and consistent teaching effect, it’s advisable for educators to conduct a preliminary survey on students’ baseline levels and optimize the SBME course.

Healthcare simulation holds the key to the future of medical education [32]. The findings from our study may carry broad implications for the medical education community and beyond. Medical educators can integrate SBME modules into their curricula and inform their own research, such as investigating the intersection of SBME with problem-based learning. The potential for scaling up the SBME curriculum is significant [32]. Our findings extended the benefits of SBME to a wider range of medical education. Educational institutions and healthcare technology companies can establish a network of simulation centers to create customized SBME modules and offer continuous support for educators, ensuring the quality and consistency of the educational experience. Healthcare regulatory bodies and policymakers can leverage our research to advocate for SBME inclusion, fostering a more standardized approach to clinical research education across institutions. This ensures that all medical students receive the essential training to conduct high-quality research.

## Strengths and limitations

Our study has the following strengths: Firstly, to the best of our knowledge, it might be the first study to coherently evaluate effectiveness of SBME for both RCT and observation study training. Secondly, the effects on both knowledge and practice were evaluated for each knowledge point in clinical research, meanwhile, influencing factors on the effectiveness was explored. Thirdly, our primary pre-post interventional study incorporated three levels of external controls (undergraduates in their 4th or 5th year, master’s and doctoral degree students), which potentially reflect the natural progression of the participants (3rd-year undergraduates) in the primary study. The inclusion of these external controls provides a comparative benchmark, facilitating the measurement of changes in the intervention group relative to a stable reference point, thereby enhancing the reliability and validity of our results.

There were several limitations should be considered in this study. Firstly, our results might be influenced by the regression to mean (RTM) effect. Based on the distribution of pre-intervention scores, there might be a few extreme measures in baseline scores. A certain correlation between pre- and post-test measurements implies that the effect of RTM might be not substantial [33]. The utilization of external controls provided a natural development that would occur without the SBME, and differences of less than 1 or no significant difference was observed between baseline scores and external controls, it suggested the true effect of the intervention instead of RTM effect. Secondly, halo effect and Hawthorne effect might influence validity of the pre-post study. Even though the external control can alleviate their bias, standard introduction of the curriculum and teachers and objective measurement of intervention effect should be developed in the further study. Due to ethical constraints, RCT was not conducted, optimal RCT can be carried out to validate the conclusion. Lastly, it was a short-term intervention effect, further longitudinal repeated measured studies could examine the long-term effect of this teaching approach on medical students’ real ability in future clinical research.

## Conclusion

In this single-arm pre-post intervention study with external controls, we observed the benefits of simulation-based clinical research curriculum which significantly improved medical students’ both knowledge and practice in clinical research. To maximize and unify the value of this innovative pedagogy, efforts should be made by medical educators to emphasize the importance of clinical research on clinical practice and develop standard teaching process. Further studies are required to explore the long-term effect of the curriculum.

### Supplementary Information

**Supplementary Material 1.**

**Supplementary Material 2.**

**Supplementary Material 3.**

## Data Availability

Dataset(s) supporting the conclusions of this article are available upon request from the first author and corresponding author.
