# Supplementary material for: Effectiveness of simulation-based clinical research curriculum for undergraduate medical students - a pre-post intervention study with external control
Source: BMC Med Educ. 2024 May 15;24:542. doi: 10.1186/s12909-024-05455-6 (PMC11097530; doi:10.1186/s12909-024-05455-6)
Supplement: Supplementary file 1 — Supplementary Material 1. [file 12909_2024_5455_MOESM1_ESM.docx]

**Supplementary file 1**

**Questionnaire on medical students’ cognition of clinical trials and scenario simulation teaching**

In the spring of 2020, the spread of Coronavirus Disease 2019 (COVID-19) pushed various R&D institutions, hospitals, and enterprises to carry out quantitative drug clinical trials, aiming to find and develop safe and effective drugs. However, problems like design, sample collecting, and analyses in some clinical trials have made it difficult to objectively and accurately evaluate their effectiveness, thus emphasizing the significance and necessity of scientific, standardized, and accurate clinical trials. The success rate of research and development can only be increased by improving the quality and level of standardization, adhering to the principles of science and norms, and making full use of precious resources.

Medical students are crucial participants in and organizers of the future’s clinical trials. Understanding their level of cognition and practical ability can help to carry out targeted teaching courses and explore efficient teaching modes, in the hope of increasing their interest, cognition, attitude, and ability. The questionnaire on medical students’ cognition of clinical trials and scenario simulation teaching held by the Second Clinical College of Wuhan University (Zhongnan Hospital) includes 3 parts: (1) basic information; (2) knowledge of and practical ability in clinical trials; (3) cognition of scenario simulation teaching. Data desensitization will be applied to protect your personal information and the answers to this questionnaire do not affect your test scores, it will take you about 15 minutes. If you agree to participate in this survey, please click “agree” and fill it out honestly. Thanks for your support!

Do you agree to take this survey?

Agree

Disagree

**Part 1: Basic Information**

**Student ID: _________________**

**Gender**: Male | Female

**Age** (18-45 years old): **_________________**

**Have you ever participated in clinical research (including clinical trial) before?**

🞏 Yes 🞏 No

(If you answer yes in last question) **What is your role in clinical research?**

🞏 Subject 🞏 Researcher

**Have you ever taken relevant systematic training in clinical trial (not regular courses, like conferences or training classes)?**

🞏 Yes 🞏 No

**Have you ever studied relevant knowledge of clinical trials on your own initiative?**

🞏 Yes 🞏 No

**Do you want to conduct a clinical trial?**

🞏 Yes 🞏 No

**Have you ever heard of scenario simulation teaching before?**

🞏 Yes 🞏 No

**To what extent do you agree or disagree with the following statements that “the increase in clinical research capabilities can improve medical staff’s clinical practice abilities”?**

| 0 | 1 | 2 | 3 | 4 | 5 | 6 | 7 | 8 | 9 | 10 |
| --- | --- | --- | --- | --- | --- | --- | --- | --- | --- | --- |
| Strongly disagree |  |  |  |  |  |  |  |  |  | Strongly agree |

**To what extent do you agree or disagree with the following statements that “clinical research can promote the development of medical science and thus benefit the patients”?**

| 0 | 1 | 2 | 3 | 4 | 5 | 6 | 7 | 8 | 9 | 10 |
| --- | --- | --- | --- | --- | --- | --- | --- | --- | --- | --- |
| Strongly disagree |  |  |  |  |  |  |  |  |  | Strongly agree |

**Part 2: Self-evaluation of knowledge and practical ability**

*Note: The following self-evaluating items are aiming at assessing knowledge and practical ability in clinical trials using a 5-point Likert scale, respectively*

| 1 | 2 | 3 | 4 | 5 |
| --- | --- | --- | --- | --- |
| Very unfamiliar | Unfamiliar | Moderate | Familiar | Very familiar |

Please fill the survey honestly reflecting your real situation, the results are only used for relevant designs of courses for the future.

**1. Please evaluate your knowledge and practical ability about clinical trial protocols.**

**1-1 About the stipulated contents and writing standards of clinical trial protocol.**

| Knowledge | 🞏 1 | 🞏 2 | 🞏 3 | 🞏 4 | 🞏 5 |
| --- | --- | --- | --- | --- | --- |
| Practice | 🞏 1 | 🞏 2 | 🞏 3 | 🞏 4 | 🞏5 |

**1-2 About the statistics of clinical trial protocol.**

| Knowledge | 🞏 1 | 🞏 2 | 🞏 3 | 🞏 4 | 🞏 5 |
| --- | --- | --- | --- | --- | --- |
| Practice | 🞏 1 | 🞏 2 | 🞏 3 | 🞏 4 | 🞏5 |

**2. Please evaluate your knowledge and practical ability about the ethics in clinical trials.**

**2-1 As a researcher, how well do you know the submission process and material submitted to Medical Ethics Committee before starting a clinical trial?**

| Knowledge | 🞏 1 | 🞏 2 | 🞏 3 | 🞏 4 | 🞏 5 |
| --- | --- | --- | --- | --- | --- |
| Practice | 🞏 1 | 🞏 2 | 🞏 3 | 🞏 4 | 🞏5 |

**2-2 As a researcher, how well do you know the submission process and material submitted to Medical Ethics Committee during the clinical trial?**

| Knowledge | 🞏 1 | 🞏 2 | 🞏 3 | 🞏 4 | 🞏 5 |
| --- | --- | --- | --- | --- | --- |
| Practice | 🞏 1 | 🞏 2 | 🞏 3 | 🞏 4 | 🞏5 |

**2-3 As a researcher, how much do you know about protecting subject’s rights and interests in clinical trials?**

| Knowledge | 🞏 1 | 🞏 2 | 🞏 3 | 🞏 4 | 🞏 5 |
| --- | --- | --- | --- | --- | --- |
| Practice | 🞏 1 | 🞏 2 | 🞏 3 | 🞏 4 | 🞏5 |

**2-4 As a researcher, how well do you know about monitoring and reporting of adverse events in clinical trials?**

| Knowledge | 🞏 1 | 🞏 2 | 🞏 3 | 🞏 4 | 🞏 5 |
| --- | --- | --- | --- | --- | --- |
| Practice | 🞏 1 | 🞏 2 | 🞏 3 | 🞏 4 | 🞏5 |

**3. Please evaluate your knowledge and practical ability about the Case Report Form (CRF).**

**3-1 designing a complete CRF**

| Knowledge | 🞏 1 | 🞏 2 | 🞏 3 | 🞏 4 | 🞏 5 |
| --- | --- | --- | --- | --- | --- |
| Practice | 🞏 1 | 🞏 2 | 🞏 3 | 🞏 4 | 🞏5 |

**3-2 the methods of transforming an original medical record to a CRF.**

| Knowledge | 🞏 1 | 🞏 2 | 🞏 3 | 🞏 4 | 🞏 5 |
| --- | --- | --- | --- | --- | --- |
| Practice | 🞏 1 | 🞏 2 | 🞏 3 | 🞏 4 | 🞏5 |

**3-3 the standard of filling, amending, and revising a CRF.**

| Knowledge | 🞏 1 | 🞏 2 | 🞏 3 | 🞏 4 | 🞏 5 |
| --- | --- | --- | --- | --- | --- |
| Practice | 🞏 1 | 🞏 2 | 🞏 3 | 🞏 4 | 🞏5 |

**3-4 About transforming paper CRF to electronic ones.**

| Knowledge | 🞏 1 | 🞏 2 | 🞏 3 | 🞏 4 | 🞏 5 |
| --- | --- | --- | --- | --- | --- |
| Practice | 🞏 1 | 🞏 2 | 🞏 3 | 🞏 4 | 🞏5 |

**3-5 About the storage of paper and electronic CRF.**

| Knowledge | 🞏 1 | 🞏 2 | 🞏 3 | 🞏 4 | 🞏 5 |
| --- | --- | --- | --- | --- | --- |
| Practice | 🞏 1 | 🞏 2 | 🞏 3 | 🞏 4 | 🞏5 |

**4. Please evaluate your knowledge and practical ability in subject recruitment and random grouping.**

**4-1 how to use computers to randomize (use computer software to generate random sequences)**

| Knowledge | 🞏 1 | 🞏 2 | 🞏 3 | 🞏 4 | 🞏 5 |
| --- | --- | --- | --- | --- | --- |
| Practice | 🞏 1 | 🞏 2 | 🞏 3 | 🞏 4 | 🞏5 |

**4-2 how to achieve blinding in clinical trials.**

| Knowledge | 🞏 1 | 🞏 2 | 🞏 3 | 🞏 4 | 🞏 5 |
| --- | --- | --- | --- | --- | --- |
| Practice | 🞏 1 | 🞏 2 | 🞏 3 | 🞏 4 | 🞏5 |

**4-3 how to maintain blinding during the research.**

| Knowledge | 🞏 1 | 🞏 2 | 🞏 3 | 🞏 4 | 🞏 5 |
| --- | --- | --- | --- | --- | --- |
| Practice | 🞏 1 | 🞏 2 | 🞏 3 | 🞏 4 | 🞏5 |

**4-4** **how to recruit subjects for clinical trials.**

| Knowledge | 🞏 1 | 🞏 2 | 🞏 3 | 🞏 4 | 🞏 5 |
| --- | --- | --- | --- | --- | --- |
| Practice | 🞏 1 | 🞏 2 | 🞏 3 | 🞏 4 | 🞏5 |

**4-5 how to communicate informed consent in clinical trials.**

| Knowledge | 🞏 1 | 🞏 2 | 🞏 3 | 🞏 4 | 🞏 5 |
| --- | --- | --- | --- | --- | --- |
| Practice | 🞏 1 | 🞏 2 | 🞏 3 | 🞏 4 | 🞏5 |

**4-6 how to screen subjects in clinical trials.**

| Knowledge | 🞏 1 | 🞏 2 | 🞏 3 | 🞏 4 | 🞏 5 |
| --- | --- | --- | --- | --- | --- |
| Practice | 🞏 1 | 🞏 2 | 🞏 3 | 🞏 4 | 🞏5 |

**5. Please evaluate your knowledge and practical skills in data management and statistical analysis.**

**5-1 the purpose and specific implementation steps of unblinding in clinical trials.**

| Knowledge | 🞏 1 | 🞏 2 | 🞏 3 | 🞏 4 | 🞏 5 |
| --- | --- | --- | --- | --- | --- |
| Practice | 🞏 1 | 🞏 2 | 🞏 3 | 🞏 4 | 🞏5 |

**5-2 formulating a complete statistical analysis plan (SAP) of a clinical trial.**

| Knowledge | 🞏 1 | 🞏 2 | 🞏 3 | 🞏 4 | 🞏 5 |
| --- | --- | --- | --- | --- | --- |
| Practice | 🞏 1 | 🞏 2 | 🞏 3 | 🞏 4 | 🞏5 |

**5-3 the establishment and management of the clinical trial database.**

| Knowledge | 🞏 1 | 🞏 2 | 🞏 3 | 🞏 4 | 🞏 5 |
| --- | --- | --- | --- | --- | --- |
| Practice | 🞏 1 | 🞏 2 | 🞏 3 | 🞏 4 | 🞏5 |

**5-4 data processing and statistical analysis of clinical trials.**

| Knowledge | 🞏 1 | 🞏 2 | 🞏 3 | 🞏 4 | 🞏 5 |
| --- | --- | --- | --- | --- | --- |
| Practice | 🞏 1 | 🞏 2 | 🞏 3 | 🞏 4 | 🞏5 |

**5-5 interpreting of statistical analysis results in clinical trials.**

| Knowledge | 🞏 1 | 🞏 2 | 🞏 3 | 🞏 4 | 🞏 5 |
| --- | --- | --- | --- | --- | --- |
| Practice | 🞏 1 | 🞏 2 | 🞏 3 | 🞏 4 | 🞏5 |

**Part 3: Post curriculum assessment**

*Please provide relevant information on this simulation teaching method in the courses you have completed.*

**To what extent do you agree or disagree that this simulation teaching can,**

| Items | 1  Strongly disagree | 2  Disagree | 3  Neutral | 4  Agree | 5  Strongly agree |
| --- | --- | --- | --- | --- | --- |
| Deepen the theoretical knowledge | 🞏 | 🞏 | 🞏 | 🞏 | 🞏 |
| Improve communication skills and abilities | 🞏 | 🞏 | 🞏 | 🞏 | 🞏 |
| Improve teamwork skills | 🞏 | 🞏 | 🞏 | 🞏 | 🞏 |
| Increase learning interest | 🞏 | 🞏 | 🞏 | 🞏 | 🞏 |
| Improve critical thinking | 🞏 | 🞏 | 🞏 | 🞏 | 🞏 |
| Improve practical skills | 🞏 | 🞏 | 🞏 | 🞏 | 🞏 |
| Improve ability to handle emergencies in research | 🞏 | 🞏 | 🞏 | 🞏 | 🞏 |

**Please assess your confidence in independently conducting clinical research in the future,**

| 0 | 1 | 2 | 3 | 4 | 5 | 6 | 7 | 8 | 9 | 10 |
| --- | --- | --- | --- | --- | --- | --- | --- | --- | --- | --- |
| No confidence |  |  |  |  |  |  |  |  |  | High confidence |

**Do you agree to use the simulation teaching in clinical research 1?**

| 🞏 1  Strongly disagree | 🞏 2  Disagree | 🞏 3  Neutral | 🞏 4  Agree | 🞏 5  Strongly agree |
| --- | --- | --- | --- | --- |
