# Supplementary material for: Effectiveness of simulation-based clinical research curriculum for undergraduate medical students - a pre-post intervention study with external control
Source: BMC Med Educ. 2024 May 15;24:542. doi: 10.1186/s12909-024-05455-6 (PMC11097530; doi:10.1186/s12909-024-05455-6)
Supplement: Supplementary file 2 — Supplementary Material 2. [file 12909_2024_5455_MOESM2_ESM.docx]

**Supplementary file 2**

**Questionnaire on medical students’ cognition of observational study and scenario simulation teaching**

Understanding the status of medical students' knowledge and practical capabilities on observational study (including cross-sectional studies, case-control studies, cohort studies, diagnostic tests, and screening, etc.) is beneficial for developing targeted educational courses and exploring effective teaching methods, finally to enhance medical students' interest, cognition, attitude, and proficiency in observational research.

The questionnaire on medical students’ cognition of observational study and scenario simulation teaching held by the Second Clinical College of Wuhan University (Zhongnan Hospital) includes 3 parts: (1) basic information; (2) knowledge of and practical ability in observational study; (3) cognition of scenario simulation teaching. Data desensitization will be applied to protect your personal information and the answers to this questionnaire do not affect your test scores, it will take you about 15 minutes. If you agree to participate in this survey, please click “agree” and fill it out honestly. Thanks for your support!

Do you agree to take this survey?

Agree

Disagree

**Part 1: Basic Information**

**Student ID: _________________**

**Gender**: Male | Female

**Age** (18-45 years old): **_________________**

**Have you ever participated in observational study before?**

🞏 Yes 🞏 No

(If you answer yes in last question) **What is your role in the observational study?**

🞏 Subject 🞏 Researcher

**Have you ever taken relevant systematic training in observational study (not regular courses, like conferences or training classes)?**

🞏 Yes 🞏 No

**Have you ever studied relevant knowledge of observational study on your own initiative?**

🞏 Yes 🞏 No

**Do you want to conduct an observational study?**

🞏 Yes 🞏 No

**To what extent do you agree or disagree with the following statements that “the increase in clinical research capabilities can improve medical staff’s clinical practice abilities”?**

| 0 | 1 | 2 | 3 | 4 | 5 | 6 | 7 | 8 | 9 | 10 |
| --- | --- | --- | --- | --- | --- | --- | --- | --- | --- | --- |
| Strongly disagree |  |  |  |  |  |  |  |  |  | Strongly agree |

**To what extent do you agree or disagree with the following statements that “clinical research can promote the development of medical science and thus benefit the patients”?**

| 0 | 1 | 2 | 3 | 4 | 5 | 6 | 7 | 8 | 9 | 10 |
| --- | --- | --- | --- | --- | --- | --- | --- | --- | --- | --- |
| Strongly disagree |  |  |  |  |  |  |  |  |  | Strongly agree |

**Part 2: Self-evaluation of knowledge and practical ability**

*Note: The following self-evaluating items are aiming at assessing knowledge and practical ability in observational study using a 5-point Likert scale, respectively*

| 1 | 2 | 3 | 4 | 5 |
| --- | --- | --- | --- | --- |
| Very unfamiliar | Unfamiliar | Moderate | Familiar | Very familiar |

Please fill the survey honestly reflecting your real situation, the results are only used for relevant designs of courses for the future.

**1. Please evaluate your knowledge and practical ability about** **observational study protocols.**

**1-1 About the stipulated contents and writing standards of observational study protocol.**

| Knowledge | 🞏 1 | 🞏 2 | 🞏 3 | 🞏 4 | 🞏 5 |
| --- | --- | --- | --- | --- | --- |
| Practice | 🞏 1 | 🞏 2 | 🞏 3 | 🞏 4 | 🞏5 |

**1-2 About the stipulated study design in observational study protocol.**

| Knowledge | 🞏 1 | 🞏 2 | 🞏 3 | 🞏 4 | 🞏 5 |
| --- | --- | --- | --- | --- | --- |
| Practice | 🞏 1 | 🞏 2 | 🞏 3 | 🞏 4 | 🞏5 |

**1-3 About the statistics of observational study protocol.**

| Knowledge | 🞏 1 | 🞏 2 | 🞏 3 | 🞏 4 | 🞏 5 |
| --- | --- | --- | --- | --- | --- |
| Practice | 🞏 1 | 🞏 2 | 🞏 3 | 🞏 4 | 🞏5 |

**2. Please evaluate your knowledge and practical ability about development of data collection tool and operational manual.**

**2-1 About design of data collection in observational study protocol, such as questionnaire.**

| Knowledge | 🞏 1 | 🞏 2 | 🞏 3 | 🞏 4 | 🞏 5 |
| --- | --- | --- | --- | --- | --- |
| Practice | 🞏 1 | 🞏 2 | 🞏 3 | 🞏 4 | 🞏5 |

**2-2 About how to develop a practical operation manual**

| Knowledge | 🞏 1 | 🞏 2 | 🞏 3 | 🞏 4 | 🞏 5 |
| --- | --- | --- | --- | --- | --- |
| Practice | 🞏 1 | 🞏 2 | 🞏 3 | 🞏 4 | 🞏5 |

**3. Please evaluate your knowledge and practical ability about pre and formal survey and recruitment.**

**3-1 How to conduct personnel training (including project managers, investigators, and auditors, etc.)**

| Knowledge | 🞏 1 | 🞏 2 | 🞏 3 | 🞏 4 | 🞏 5 |
| --- | --- | --- | --- | --- | --- |
| Practice | 🞏 1 | 🞏 2 | 🞏 3 | 🞏 4 | 🞏5 |

**3-2 Purpose and conduct of a preliminary investigation**

| Knowledge | 🞏 1 | 🞏 2 | 🞏 3 | 🞏 4 | 🞏 5 |
| --- | --- | --- | --- | --- | --- |
| Practice | 🞏 1 | 🞏 2 | 🞏 3 | 🞏 4 | 🞏5 |

**3-3 Recruitment of participants**

| Knowledge | 🞏 1 | 🞏 2 | 🞏 3 | 🞏 4 | 🞏 5 |
| --- | --- | --- | --- | --- | --- |
| Practice | 🞏 1 | 🞏 2 | 🞏 3 | 🞏 4 | 🞏5 |

**3-4 Process of formal survey**

| Knowledge | 🞏 1 | 🞏 2 | 🞏 3 | 🞏 4 | 🞏 5 |
| --- | --- | --- | --- | --- | --- |
| Practice | 🞏 1 | 🞏 2 | 🞏 3 | 🞏 4 | 🞏5 |

**3-5 Designing and filling out required forms for formal investigations (e.g., registration form, appointment letter, informed consent)**

| Knowledge | 🞏 1 | 🞏 2 | 🞏 3 | 🞏 4 | 🞏 5 |
| --- | --- | --- | --- | --- | --- |
| Practice | 🞏 1 | 🞏 2 | 🞏 3 | 🞏 4 | 🞏5 |

**4. Please evaluate your knowledge and practical ability in data collection and verification.**

**4-1 Establishment of databases and data collection methods in observational study.**

| Knowledge | 🞏 1 | 🞏 2 | 🞏 3 | 🞏 4 | 🞏 5 |
| --- | --- | --- | --- | --- | --- |
| Practice | 🞏 1 | 🞏 2 | 🞏 3 | 🞏 4 | 🞏5 |

**4-2 Date check and verification in observational study.**

| Knowledge | 🞏 1 | 🞏 2 | 🞏 3 | 🞏 4 | 🞏 5 |
| --- | --- | --- | --- | --- | --- |
| Practice | 🞏 1 | 🞏 2 | 🞏 3 | 🞏 4 | 🞏5 |

**5. Please evaluate your knowledge and practical skills in and statistical analysis.**

**5-1 Statistical analysis in observational study.**

| Knowledge | 🞏 1 | 🞏 2 | 🞏 3 | 🞏 4 | 🞏 5 |
| --- | --- | --- | --- | --- | --- |
| Practice | 🞏 1 | 🞏 2 | 🞏 3 | 🞏 4 | 🞏5 |

**5-2 Interpreting of statistical analysis results in observational study.**

| Knowledge | 🞏 1 | 🞏 2 | 🞏 3 | 🞏 4 | 🞏 5 |
| --- | --- | --- | --- | --- | --- |
| Practice | 🞏 1 | 🞏 2 | 🞏 3 | 🞏 4 | 🞏5 |

**Part 3: Post curriculum assessment**

*Please provide relevant information on this simulation teaching method in the courses you have completed.*

**To what extent do you agree or disagree that this simulation teaching can,**

| Items | 1  Strongly disagree | 2  Disagree | 3  Neutral | 4  Agree | 5  Strongly agree |
| --- | --- | --- | --- | --- | --- |
| Deepen the theoretical knowledge | 🞏 | 🞏 | 🞏 | 🞏 | 🞏 |
| Improve communication skills and abilities | 🞏 | 🞏 | 🞏 | 🞏 | 🞏 |
| Improve teamwork skills | 🞏 | 🞏 | 🞏 | 🞏 | 🞏 |
| Increase learning interest | 🞏 | 🞏 | 🞏 | 🞏 | 🞏 |
| Improve critical thinking | 🞏 | 🞏 | 🞏 | 🞏 | 🞏 |
| Improve practical skills | 🞏 | 🞏 | 🞏 | 🞏 | 🞏 |
| Improve ability to handle emergencies in research | 🞏 | 🞏 | 🞏 | 🞏 | 🞏 |

**Please assess your confidence in independently conducting an observational study in the future,**

| 0 | 1 | 2 | 3 | 4 | 5 | 6 | 7 | 8 | 9 | 10 |
| --- | --- | --- | --- | --- | --- | --- | --- | --- | --- | --- |
| No confidence |  |  |  |  |  |  |  |  |  | High confidence |

**Do you agree to use the simulation teaching in clinical research 1?**

| 🞏 1  Strongly disagree | 🞏 2  Disagree | 🞏 3  Neutral | 🞏 4  Agree | 🞏 5  Strongly agree |
| --- | --- | --- | --- | --- |
