# Supplementary material for: Effectiveness of simulation-based clinical research curriculum for undergraduate medical students - a pre-post intervention study with external control
Source: BMC Med Educ. 2024 May 15;24:542. doi: 10.1186/s12909-024-05455-6 (PMC11097530; doi:10.1186/s12909-024-05455-6)
Supplement: Supplementary file 3 — Supplementary Material 3. [file 12909_2024_5455_MOESM3_ESM.docx]

**Supplementary file 3**

**A cross sectional survey on medical students’ cognition of clinical research**

Clinical research is a vital component of medical studies and a significant reflection of medical students' learning and research abilities. Clinical students are essential organizers or participants in future clinical research. In-depth study of clinical research methodology and skills can enhance academic literacy, foster critical thinking and analytical abilities, and enable a more scientific and systematic approach to diseases, leading to accurate diagnosis and treatment decisions in future clinical practice.

Understanding the current state of clinical students' awareness and proficiency in clinical research can help develop targeted teaching courses, explore efficient teaching models, and ultimately increase clinical students' interest, awareness, attitude, and competence in clinical research. Wuhan University's Second Clinical College conducted a "Survey on Clinical Students' Awareness and Abilities in Clinical Research", which consists of three parts: (1) Basic Information; (2) Understanding and Operation of Clinical Trials; (3) Understanding and Operation of Observational Studies. By clicking "Agree", you consent to participate in this survey. Your personal information will be de-identified, and the survey results are unrelated to exam scores, with an estimated completion time of approximately 20 minutes.

Do you agree to take this survey?

Agree

Disagree

**Part 1: Basic Information**

**Gender**: Male | Female

**Age** (18-45 years old): **_________________**

**Have you ever participated in clinical research (including clinical trial) before?**

🞏 Yes 🞏 No

(If you answer yes in last question) **What is your role in clinical** **research?**

🞏 Subject 🞏 Researcher

**Have you ever taken relevant systematic training in clinical research (not regular courses, like conferences or training classes)?**

🞏 Yes 🞏 No

**To what extent do you agree or disagree with the following statements that “the increase in clinical research capabilities can improve medical staff’s clinical practice abilities”?**

| 0 | 1 | 2 | 3 | 4 | 5 | 6 | 7 | 8 | 9 | 10 |
| --- | --- | --- | --- | --- | --- | --- | --- | --- | --- | --- |
| Strongly disagree |  |  |  |  |  |  |  |  |  | Strongly agree |

**To what extent do you agree or disagree with the following statements that “clinical research can promote the development of medical science and thus benefit the patients”?**

| 0 | 1 | 2 | 3 | 4 | 5 | 6 | 7 | 8 | 9 | 10 |
| --- | --- | --- | --- | --- | --- | --- | --- | --- | --- | --- |
| Strongly disagree |  |  |  |  |  |  |  |  |  | Strongly agree |

**Part 2: Self-evaluation of knowledge and practice in clinical trial**

*Note: The following self-evaluating items are aiming at assessing knowledge and practical ability in clinical trials using a 5-point Likert scale, respectively*

| 1 | 2 | 3 | 4 | 5 |
| --- | --- | --- | --- | --- |
| Very unfamiliar | Unfamiliar | Moderate | Familiar | Very familiar |

Please fill the survey honestly reflecting your real situation, the results are only used for relevant designs of courses for the future.

**1. Please evaluate your knowledge and practical ability about clinical trial protocols.**

**1-1 About the stipulated contents and writing standards of clinical trial protocol.**

| Knowledge | 🞏 1 | 🞏 2 | 🞏 3 | 🞏 4 | 🞏 5 |
| --- | --- | --- | --- | --- | --- |
| Practice | 🞏 1 | 🞏 2 | 🞏 3 | 🞏 4 | 🞏5 |

**1-2 About the statistics of clinical trial protocol.**

| Knowledge | 🞏 1 | 🞏 2 | 🞏 3 | 🞏 4 | 🞏 5 |
| --- | --- | --- | --- | --- | --- |
| Practice | 🞏 1 | 🞏 2 | 🞏 3 | 🞏 4 | 🞏5 |

**2. Please evaluate your knowledge and practical ability about the ethics in clinical trials.**

**2-1 As a researcher, how well do you know the submission process and material submitted to Medical Ethics Committee before starting a clinical trial?**

| Knowledge | 🞏 1 | 🞏 2 | 🞏 3 | 🞏 4 | 🞏 5 |
| --- | --- | --- | --- | --- | --- |
| Practice | 🞏 1 | 🞏 2 | 🞏 3 | 🞏 4 | 🞏5 |

**2-2 As a researcher, how well do you know the submission process and material submitted to Medical Ethics Committee during the clinical trial?**

| Knowledge | 🞏 1 | 🞏 2 | 🞏 3 | 🞏 4 | 🞏 5 |
| --- | --- | --- | --- | --- | --- |
| Practice | 🞏 1 | 🞏 2 | 🞏 3 | 🞏 4 | 🞏5 |

**2-3 As a researcher, how much do you know about protecting subject’s rights and interests in clinical trials?**

| Knowledge | 🞏 1 | 🞏 2 | 🞏 3 | 🞏 4 | 🞏 5 |
| --- | --- | --- | --- | --- | --- |
| Practice | 🞏 1 | 🞏 2 | 🞏 3 | 🞏 4 | 🞏5 |

**2-4 As a researcher, how well do you know about monitoring and reporting of adverse events in clinical trials?**

| Knowledge | 🞏 1 | 🞏 2 | 🞏 3 | 🞏 4 | 🞏 5 |
| --- | --- | --- | --- | --- | --- |
| Practice | 🞏 1 | 🞏 2 | 🞏 3 | 🞏 4 | 🞏5 |

**3. Please evaluate your knowledge and practical ability about statistical analysis in observational study.**

**3-1 designing a complete CRF**

| Knowledge | 🞏 1 | 🞏 2 | 🞏 3 | 🞏 4 | 🞏 5 |
| --- | --- | --- | --- | --- | --- |
| Practice | 🞏 1 | 🞏 2 | 🞏 3 | 🞏 4 | 🞏5 |

**3-2 the methods of transforming an original medical record to a CRF.**

| Knowledge | 🞏 1 | 🞏 2 | 🞏 3 | 🞏 4 | 🞏 5 |
| --- | --- | --- | --- | --- | --- |
| Practice | 🞏 1 | 🞏 2 | 🞏 3 | 🞏 4 | 🞏5 |

**3-3 the standard of filling, amending, and revising a CRF.**

| Knowledge | 🞏 1 | 🞏 2 | 🞏 3 | 🞏 4 | 🞏 5 |
| --- | --- | --- | --- | --- | --- |
| Practice | 🞏 1 | 🞏 2 | 🞏 3 | 🞏 4 | 🞏5 |

**3-4 About transforming paper CRF to electronic ones.**

| Knowledge | 🞏 1 | 🞏 2 | 🞏 3 | 🞏 4 | 🞏 5 |
| --- | --- | --- | --- | --- | --- |
| Practice | 🞏 1 | 🞏 2 | 🞏 3 | 🞏 4 | 🞏5 |

**3-5 About the storage of paper and electronic CRF.**

| Knowledge | 🞏 1 | 🞏 2 | 🞏 3 | 🞏 4 | 🞏 5 |
| --- | --- | --- | --- | --- | --- |
| Practice | 🞏 1 | 🞏 2 | 🞏 3 | 🞏 4 | 🞏5 |

**4. Please evaluate your knowledge and practical ability in subject recruitment and random grouping.**

**4-1 how to use computers to randomize (use computer software to generate random sequences)**

| Knowledge | 🞏 1 | 🞏 2 | 🞏 3 | 🞏 4 | 🞏 5 |
| --- | --- | --- | --- | --- | --- |
| Practice | 🞏 1 | 🞏 2 | 🞏 3 | 🞏 4 | 🞏5 |

**4-2 how to achieve blinding in clinical trials.**

| Knowledge | 🞏 1 | 🞏 2 | 🞏 3 | 🞏 4 | 🞏 5 |
| --- | --- | --- | --- | --- | --- |
| Practice | 🞏 1 | 🞏 2 | 🞏 3 | 🞏 4 | 🞏5 |

**4-3 how to maintain blinding during the research.**

| Knowledge | 🞏 1 | 🞏 2 | 🞏 3 | 🞏 4 | 🞏 5 |
| --- | --- | --- | --- | --- | --- |
| Practice | 🞏 1 | 🞏 2 | 🞏 3 | 🞏 4 | 🞏5 |

**4-4** **how to recruit subjects for clinical trials.**

| Knowledge | 🞏 1 | 🞏 2 | 🞏 3 | 🞏 4 | 🞏 5 |
| --- | --- | --- | --- | --- | --- |
| Practice | 🞏 1 | 🞏 2 | 🞏 3 | 🞏 4 | 🞏5 |

**4-5 how to communicate informed consent in clinical trials.**

| Knowledge | 🞏 1 | 🞏 2 | 🞏 3 | 🞏 4 | 🞏 5 |
| --- | --- | --- | --- | --- | --- |
| Practice | 🞏 1 | 🞏 2 | 🞏 3 | 🞏 4 | 🞏5 |

**4-6 how to screen subjects in clinical trials.**

| Knowledge | 🞏 1 | 🞏 2 | 🞏 3 | 🞏 4 | 🞏 5 |
| --- | --- | --- | --- | --- | --- |
| Practice | 🞏 1 | 🞏 2 | 🞏 3 | 🞏 4 | 🞏5 |

**5. Please evaluate your knowledge and practical skills in data management and statistical analysis.**

**5-1 the purpose and specific implementation steps of unblinding in clinical trials.**

| Knowledge | 🞏 1 | 🞏 2 | 🞏 3 | 🞏 4 | 🞏 5 |
| --- | --- | --- | --- | --- | --- |
| Practice | 🞏 1 | 🞏 2 | 🞏 3 | 🞏 4 | 🞏5 |

**5-2 formulating a complete statistical analysis plan (SAP) of a clinical trial.**

| Knowledge | 🞏 1 | 🞏 2 | 🞏 3 | 🞏 4 | 🞏 5 |
| --- | --- | --- | --- | --- | --- |
| Practice | 🞏 1 | 🞏 2 | 🞏 3 | 🞏 4 | 🞏5 |

**5-3 the establishment and management of the clinical trial database.**

| Knowledge | 🞏 1 | 🞏 2 | 🞏 3 | 🞏 4 | 🞏 5 |
| --- | --- | --- | --- | --- | --- |
| Practice | 🞏 1 | 🞏 2 | 🞏 3 | 🞏 4 | 🞏5 |

**5-4 data processing and statistical analysis of clinical trials.**

| Knowledge | 🞏 1 | 🞏 2 | 🞏 3 | 🞏 4 | 🞏 5 |
| --- | --- | --- | --- | --- | --- |
| Practice | 🞏 1 | 🞏 2 | 🞏 3 | 🞏 4 | 🞏5 |

**5-5 interpreting of statistical analysis results in clinical trials.**

| Knowledge | 🞏 1 | 🞏 2 | 🞏 3 | 🞏 4 | 🞏 5 |
| --- | --- | --- | --- | --- | --- |
| Practice | 🞏 1 | 🞏 2 | 🞏 3 | 🞏 4 | 🞏5 |

**Part 3: Self-evaluation of knowledge and practical ability in**

**observational study**

*Note: The following self-evaluating items are aiming at assessing knowledge and practical ability in observational study using a 5-point Likert scale, respectively*

| 1 | 2 | 3 | 4 | 5 |
| --- | --- | --- | --- | --- |
| Very unfamiliar | Unfamiliar | Moderate | Familiar | Very familiar |

Please fill the survey honestly reflecting your real situation, the results are only used for relevant designs of courses for the future.

**1. Please evaluate your knowledge and practical ability about observational study protocols.**

**1-1 About the stipulated contents and writing standards of observational study protocol.**

| Knowledge | 🞏 1 | 🞏 2 | 🞏 3 | 🞏 4 | 🞏 5 |
| --- | --- | --- | --- | --- | --- |
| Practice | 🞏 1 | 🞏 2 | 🞏 3 | 🞏 4 | 🞏5 |

**1-2 About the stipulated study design in observational study protocol.**

| Knowledge | 🞏 1 | 🞏 2 | 🞏 3 | 🞏 4 | 🞏 5 |
| --- | --- | --- | --- | --- | --- |
| Practice | 🞏 1 | 🞏 2 | 🞏 3 | 🞏 4 | 🞏5 |

**1-3 About the statistics of observational study protocol.**

| Knowledge | 🞏 1 | 🞏 2 | 🞏 3 | 🞏 4 | 🞏 5 |
| --- | --- | --- | --- | --- | --- |
| Practice | 🞏 1 | 🞏 2 | 🞏 3 | 🞏 4 | 🞏5 |

**2. Please evaluate your knowledge and practical ability about development of data collection tool and operational manual.**

**2-1 About design of data collection in observational study protocol, such as questionnaire.**

| Knowledge | 🞏 1 | 🞏 2 | 🞏 3 | 🞏 4 | 🞏 5 |
| --- | --- | --- | --- | --- | --- |
| Practice | 🞏 1 | 🞏 2 | 🞏 3 | 🞏 4 | 🞏5 |

**2-2 About how to develop a practical operation manual**

| Knowledge | 🞏 1 | 🞏 2 | 🞏 3 | 🞏 4 | 🞏 5 |
| --- | --- | --- | --- | --- | --- |
| Practice | 🞏 1 | 🞏 2 | 🞏 3 | 🞏 4 | 🞏5 |

**3. Please evaluate your knowledge and practical ability about pre and formal survey and recruitment.**

**3-1 How to conduct personnel training (including project managers, investigators, and auditors, etc.)**

| Knowledge | 🞏 1 | 🞏 2 | 🞏 3 | 🞏 4 | 🞏 5 |
| --- | --- | --- | --- | --- | --- |
| Practice | 🞏 1 | 🞏 2 | 🞏 3 | 🞏 4 | 🞏5 |

**3-2 Purpose and conduct of a preliminary investigation**

| Knowledge | 🞏 1 | 🞏 2 | 🞏 3 | 🞏 4 | 🞏 5 |
| --- | --- | --- | --- | --- | --- |
| Practice | 🞏 1 | 🞏 2 | 🞏 3 | 🞏 4 | 🞏5 |

**3-3 Recruitment of participants**

| Knowledge | 🞏 1 | 🞏 2 | 🞏 3 | 🞏 4 | 🞏 5 |
| --- | --- | --- | --- | --- | --- |
| Practice | 🞏 1 | 🞏 2 | 🞏 3 | 🞏 4 | 🞏5 |

**3-4 Process of formal survey**

| Knowledge | 🞏 1 | 🞏 2 | 🞏 3 | 🞏 4 | 🞏 5 |
| --- | --- | --- | --- | --- | --- |
| Practice | 🞏 1 | 🞏 2 | 🞏 3 | 🞏 4 | 🞏5 |

**3-5 Designing and filling out required forms for formal investigations (e.g., registration form, appointment letter, informed consent)**

| Knowledge | 🞏 1 | 🞏 2 | 🞏 3 | 🞏 4 | 🞏 5 |
| --- | --- | --- | --- | --- | --- |
| Practice | 🞏 1 | 🞏 2 | 🞏 3 | 🞏 4 | 🞏5 |

**4. Please evaluate your knowledge and practical ability in data collection and verification.**

**4-1 Establishment of databases and data collection methods in observational study.**

| Knowledge | 🞏 1 | 🞏 2 | 🞏 3 | 🞏 4 | 🞏 5 |
| --- | --- | --- | --- | --- | --- |
| Practice | 🞏 1 | 🞏 2 | 🞏 3 | 🞏 4 | 🞏5 |

**4-2 Date check and verification in observational study.**

| Knowledge | 🞏 1 | 🞏 2 | 🞏 3 | 🞏 4 | 🞏 5 |
| --- | --- | --- | --- | --- | --- |
| Practice | 🞏 1 | 🞏 2 | 🞏 3 | 🞏 4 | 🞏5 |

**5. Please evaluate your knowledge and practical skills in and statistical analysis.**

**5-1 Statistical analysis in observational study.**

| Knowledge | 🞏 1 | 🞏 2 | 🞏 3 | 🞏 4 | 🞏 5 |
| --- | --- | --- | --- | --- | --- |
| Practice | 🞏 1 | 🞏 2 | 🞏 3 | 🞏 4 | 🞏5 |

**5-2 Interpreting of statistical analysis results in observational study.**

| Knowledge | 🞏 1 | 🞏 2 | 🞏 3 | 🞏 4 | 🞏 5 |
| --- | --- | --- | --- | --- | --- |
| Practice | 🞏 1 | 🞏 2 | 🞏 3 | 🞏 4 | 🞏5 |
